# Urine Metabolomic Signature of People Diagnosed with Balkan Endemic Nephropathy and Other Types of Chronic Kidney Disease Compared with Healthy Subjects in Romania

**DOI:** 10.3390/metabo13050609

**Published:** 2023-04-28

**Authors:** Valentin L. Ordodi, Nicoleta G. Hădărugă, Daniel I. Hădărugă, Alexandra T. Lukinich-Gruia, Mihaela Mărgineanu, Călin A. Tatu, Virgil Păunescu

**Affiliations:** 1 Department of Applied Chemistry, Organic and Natural Compounds Engineering, Polytechnic University of Timisoara, Carol Telbisz 6, 300001 Timisoara, Romania; valentin.ordodi@upt.ro (V.L.O.); daniel.hadaruga@upt.ro (D.I.H.); 2 Department of Food Science, Banat University of Agricultural Sciences and Veterinary Medicine Timisoara, Calea Aradului 119, 300645 Timisoara, Romania; nicoletahadaruga@usab-tm.ro; 3 Centre for Gene and Cellular Therapies in the Treatment of Cancer—OncoGen, Clinical County Hospital Timisoara, Blvd. Liviu Rebreanu 156, 300736 Timisoara, Romania; geomed88@gmail.com (C.A.T.); vpaunescu@umft.ro (V.P.); 4 Dialysis Center Fresenius NephroCare, 220012 Drobeta-Turnu Severin, Romania; 5 Department of Functional Sciences, “Victor Babes” University of Medicine and Pharmacy Timisoara, 300041 Timisoara, Romania

**Keywords:** Romanian Balkan endemic nephropathy, untargeted gas-chromatography mass spectrometry, urine biomarkers, multivariate analysis, discriminatory metabolites

## Abstract

Metabolomic analysis methods were employed to determine biomarkers for various chronic kidney diseases (CKDs). Modern analytical methods were developed and applied successfully to find a specific metabolomic profile in urine samples from CKD and Balkan endemic nephropathy (BEN) patients. The aim was to explore a specific metabolomic profile defined by feasible/easy-to-identify molecular markers. Urine samples were collected from patients with CKDs and BEN, and from healthy subjects from endemic and nonendemic areas in Romania. Metabolomic analysis of urine samples, extracted by the liquid-liquid extraction (LLE) method, was performed by gas chromatography-mass spectrometry (GC-MS). The statistical exploration of the results was performed through a principal component analysis (PCA) evaluation. Urine samples were statistically analyzed using a classification based on six types of metabolites. Most urinary metabolites are distributed in the center of a loading plot, meaning that these compounds do not represent significant markers for BEN. One of the most frequent and higher-concentration urinary metabolites in BEN patients was p-Cresol, a phenolic compound that implies a severe injury of the renal filtration function. The presence of p-Cresol was associated with protein-bound uremic toxins, which have specific functional groups such as indole and phenyl. In prospective studies for future investigation, prevention, and disease treatment, we suggest a larger sample size, sample extraction using other methods, and analysis using other chromatography techniques coupled with mass spectrometry, which can generate a more significant data set for statistical analysis.

## 1. Introduction

Chronic kidney diseases (CKDs) affect almost 15% of the world population and are considered an emerging global health problem [1]. The most puzzling CKDs have an unknown or multifactorial etiology [1]. One of these diseases is Balkan endemic nephropathy (BEN), first discovered in the 1950s, a slow, progressive kidney disease that can be discovered only in its chronic phase with end-stage renal failure and is manifested in rural communities in countries in the Balkan Peninsula, such as Romania, Serbia, Bulgaria, Croatia, and Bosnia and Herzegovina [2]. Many studies have looked for etiological factors of this disease, and many theories have been developed. The current general consensus is that aristolochic acid I (AAI), a potent carcinogenic nephrotoxin, is involved to some extent in disease causation, and also in other CKDs such as aristolochic acid nephropathy (AAN) [3,4]. However, so far, specific biomarkers for the early stage of kidney damage have been poorly defined [5], and the disease can be diagnosed only when some clinical features emerge and extensive tubular damage and urothelial tumors become manifest. The disease is typically discovered in its late, irreversible stage, after the age of 40, and is often fatal in the absence of hemodialysis or kidney transplant. Because it begins to manifest around midlife and has no specific causal factors, there are no prevention measures that can be recommended [6]. Understanding the pathophysiology and identification of diagnostic biomarkers is essential for appropriate and timely management of this complex disease [7].

Urine-based metabolomics has been applied in research on early detection, renal toxicity, diagnosis, biomarker identification, and pathogenic pathways of kidney disorders and kidney cancer [8]. One analytical method employed in the metabolomics field for urine analysis is gas chromatography-mass spectrometry (GC-MS), which separates urine metabolites specifically and more efficiently [7,9]. One of the most common and nonselective methods, which allows identification of all urine metabolites, is untargeted GC-MS analysis [9,10]. GC-MS is a powerful analytical platform for identifying, noninvasively and at minimum cost, large numbers of structures of candidate biomarkers [5,8]. With the help of GC-MS, small volumes of urine can offer results that lead to possible recognition of a disorder based on combinations of various common biomarkers, even in earlier stages, thus conferring predictive power [7,9]. In addition to GC-MS analysis, another fast and low-cost procedure through which an increased number of uncharacterized compounds can be detected is the simple liquid-liquid extraction (LLE) method [10,11]. The LLE method is frequently employed to obtain large numbers of compounds because it is a low-cost and easy-to-use screening method. One of the most commonly utilized solvents for LLE is dichloromethane (DCM), which can extract metabolites with a wide range of polarities, especially nonpolar compounds that are immiscible with water [12]. Because of specific conditions of GC-MS, sample preparation can produce volatile compounds (VCs) [5]. VCs and low-molecular-weight (molecular weight (MW) ≤ 1500 Da) metabolites in urine encompass different chemical classes, such as alcohols, organic acids, aldehydes, ketones, free fatty acids, sugars, citric acid cycle metabolites, and compounds with sulfur; all these compounds can have different concentrations, polarities, and volatilities and can be analyzed without derivatization [5,9]. The analyzed metabolites may vary due to pathological state, exposure to environmental pollutants, and diet, which are changes that can be evaluated through modern analytical screening methods [5]. Various compounds produced in cells, and their presence in different numbers, amounts and profiles in urine depend on metabolic changes, which could highlight a possible disease such as a kidney impairment condition [5]. Interpretation of the results obtained from untargeted metabolome studies using mass spectrometry is based on various statistical methods [13]. When choosing the appropriate statistical method, the characteristics of the dataset should be considered [14]. One method for analyzing the results is principal component analysis (PCA), through which the dimensionality of data sets is reduced and data variability can be explained and visualized [15].

In countries such as Romania, there are no screening methods based on metabolomics for BEN or other CKDs. Usually, clinical and paraclinical investigations are performed in response to requests from patients, making it more challenging for the disease to be diagnosed in the early stages, when the therapeutic approaches would be more effective. General evaluation of Romanian patients with BEN is performed in only one clinical center, Renamed in Mehedinti County (southwestern Romania, in the epicenter of the endemic area), where these patients are also treated by hemodialysis. Metabolomics could help provide valuable information about metabolic processes and their imbalances manifested in BEN. Although BEN was identified more than 70 years ago, complex investigations regarding its prevention have not been performed, and there are very few studies on changes in urinary metabolites. Some of the urine metabolites associated with BEN, such as β_2_-microglobulin, creatinine, urea cycle metabolites, and uric acid, were determined by nuclear magnetic resonance (NMR) [16]. Another study, performed by Trnacevic et al. (2017) in Bosnia and Herzegovina, investigated early screening of this disease, elevated microalbuminuria being considered the only reliable biomarker for in-field detection of BEN [17].

Our study aimed to employ a cheap DCM-based LLE technique followed by the simple, reliable, and sensitive GC-MS analytical method. This screening procedure was used to identify volatile compounds in urine, and we also looked for the presence of one of the most common factors of BEN and other types of CKDs (e.g., AAN), aristolochic acid I. All results were grouped to obtain specific urinary markers using PCA, a simple but powerful statistical method. These evaluation methods were chosen to obtain a quick and general result from a limited number of patients. However, further metabolomic investigations are necessary to study a disease with such multifactorial etiology at a larger sample scale and validate the potential biomarkers for clinical diagnosis and ethiopathogenic mechanism investigation.

## 2. Materials and Methods

### 2.1. Chemicals and Solvents

The following commercial analytical chemicals were used: anhydrous sodium sulphate (ACS reagent, >99.9%), DCM [Pesticide Residue Analysis (PRA) purity, >99.98%], and aristolochic acid I [High Performance Liquid Chromatography (HPLC) purity, ≥90%]. These were purchased from Sigma-Aldrich (St. Louis, MO, USA).

### 2.2. Demographic Characteristics

A general overview of the study population is presented in Table 1.

### 2.3. Urine Sample Collection

Morning midstream urine samples were collected from informed volunteers, and written consent was obtained before sample collection. The collected samples (~10 mL) were mixed with a solution of 1% sodium azide prepared in sterile deionized water to inhibit bacterial growth, and stored at 4 °C for 2 to 4 h (until extraction). The urine samples were then extracted (as described in Section 2.4) and stored at −20 °C until analysis. The preservation and storage methods did not affect the metabolite content of the urine samples. Procedures involving human participants were performed following ethical standards. All data and human sample collection was performed before the present study commenced.

(1) Healthy control volunteers (*n* = 35; code names 23, 25, 28–36, 38–61) who are family members of BEN patients (*n* = 8) or live in BEN areas (“endemic controls”) (*n* = 27); these were self-selected by availability. We ensured that only people without any documented history of kidney disease and not currently taking prescribed medication participated in our study. Urine samples (0.66 male/female ratio) collected during the first part of the day served as controls for the samples collected from the BEN patients.
(2) Patients with nephrological diseases other than BEN (*n* = 5; code names C1, C2, C3, C4, and C5) registered at the Clinical County Hospital, Timisoara, Romania, served as disease controls (1.5 male/female ratio).
(3) Patients diagnosed with BEN (*n* = 25; code names 1–22, 24, 26, and 27) under hemodialysis at Renamed Centre, Drobeta-Turnu Severin, Mehedinti County. Patients (0.71 male/female ratio) were residents of Erghevita, Bistrita and Valea Izvorului villages in Mehedinti County. The subjects were self-selected during visits for hemodialysis sessions performed at the clinic, and none was suspected to have urinary tract tumors.

### 2.4. Urine Sample Extraction

All glassware was rinsed three times with acetone and DCM and dried to minimize contamination. The anhydrous sodium sulphate was also rinsed with DCM to eliminate any chemical residue. Urine samples were subjected to the following liquid-liquid extraction (LLE) protocol: 2 mL urine was extracted with 4 mL DCM. This mixture was left to a continuous extraction process in 10 mL tubes on a horizontal shaker for 24 h at room temperature. As a result of the separatory process, two liquid phases were formed: the upper phase contained the aqueous urine phase, and the lower phase contained the organic DCM (solvent) phase. After the complete separation of the two phases, the lower one was transferred to another tube and evaporated to dryness under inert nitrogen gas, and the dry residue was kept at −20 °C until analysis.

### 2.5. Gas Chromatography-Mass Spectrometry (GC-MS) Analysis

Prior to injection into the GC-MS system, the final residue obtained in the previous stage was restored in 50 µL of PRA grade DCM after reaching room temperature. Then 2 µL of the extract was injected manually in splitless mode into the inlet, which was heated at a temperature of 180 °C. Three different concentrations of urine samples spiked with aristolochic acid I were prepared in DCM, yielding final molar concentrations of 1.03 µM, 0.20 µM and 0.02 µM. The GC-MS conditions were established as follows: the injected sample was passed through a non-polar capillary column (Agilent HP-5MS, 30 m × 0.25 mm × 0.25 µm) carried by a helium flow rate of 1 mL/min. The temperature program of the oven was set to increase from 50 to 250 °C at a heating rate of 6 °C/min and a final hold of 5 min. The conditions of the mass spectrometer system were: source temperature set at 230 °C, quadrupole detector temperature set at 150 °C, and electron ionization (EI) mode of 70 eV. The mass range of detected compounds was from 50 to 550 amu, registered after 3 min of solvent delay. Peaks were integrated using the MS software Chemstation Data Analysis (Agilent Technologies, Santa Clara, CA, USA) and AMDIS (NIST, Gaithersburg, MD, USA), and mass fragments specific for aristolochic acid I were identified in urine samples using a spiked urine sample as a standard. All chromatograms were processed with Enhanced MSD ChemStation software ver.D.02.00/2005 (Agilent Technologies, Santa Clara, CA, USA) and AMDIS GC/MS Analysis software version 2.70. Deconvoluted spectra obtained by the specific data processing protocol were introduced into the Agilent ChemStation and AMDIS GC/MS Analysis search software, and an automatic search of compound information from the NIST MS Search 98 and 2.0 libraries (NIST, Gaithersburg, MD, USA) was performed. Search results with match similarity above 90% were accepted as candidate compounds, and their mass spectra were compared to those obtained from the MS libraries. The relative concentrations calculated with Chemstation Data Analysis software were used for statistical evaluations.

### 2.6. Principal Component Analysis (PCA) and Classical Statistical Analysis

PCA is a powerful multivariate analysis technique able to extract useful information from a large dataset. This technique provides a lower dimensional principal subspace that consists of orthogonal principal components (PCs, the new uncorrelated variables). PC_1_ is obtained as the direction in the original property space, which provides maximum covariance of the projected data. PC_2_ is obtained in the same manner, but with the restriction of orthogonality to the PC_1_, and so on. As a consequence, these PCs are linear combinations of the original variables. In terms of linear algebra, PCA is an approximation of the dataset, defining the X matrix as a product of two matrices with reduced dimensionality, T and P. The first matrix provides a score plot by plotting its columns, while the second matrix is obtained by plotting its rows, resulting in the loading plot. The score plot provides information about the grouping of cases, represented by the BEN patients, patients with other types of CKDs, and control volunteers. The loading plot provides information about the influence of variables on this grouping, represented by the urinary metabolites. The first PCs will be sufficient to explain the main variance of the data. In order to reduce bias, the original data are centered and normalized [18,19]. In our study, a total of 65 cases and 262 variables were used for PCA analysis. The analysis was performed using Unscrambler 6 (CAMO, Trondheim, Norway). The cross-validation method and centered data were considered. Before PCA statistical analysis, duplicate compounds were removed and only the relative concentrations were considered for calculations. Based on the urinary metabolites obtained from GC-MS analysis, a statistical evaluation was made with all cases versus metabolites, but samples were also grouped into six types, Group 1 to Group 6. Classification was performed by grouping the metabolites into six main classes: (1) drug metabolites; (2) phenolic compounds; (3) hormone metabolites; (4) metabolites other than drug metabolites; (5) metabolites without p-Cresol; and (6) metabolites of BEN patients and volunteer (endemic area) controls, represented by their family members.

## 3. Results

The 65 subjects included in the present study were males and females aged between 58 and 75 and living in villages in Mehedinti County, Romania. A total of 262 compounds were identified in urine samples in several main classes; most of them were miscellaneous, having more than one reactive group. Compound appearance in a chromatogram is based on mass-to-charge (*m*/*z*) ratio and polarity. The classes of compounds are: aliphatic and aromatic acids, fatty acids, amines, amides, sterols, alkanes, alkenes, alcohols, phenols, esters, aldehydes, ketones, hydroxides, indoles, pyrazines, pyridines, xanthines, carbohydrates, phenyl compounds, and carboxylic acids (Table S1). Because aristolochic acids are considered one of the major factors involved in BEN, the specific fragmentation ions of aristolochic acid I were also included in the search algorithm. Therefore, the aristolochic acid I standard was injected under the same conditions as the urine samples; ions resulting from the EI fragmentation were 264 *m*/*z*, 279 *m*/*z* and 294 *m*/*z*, respectively, with a mass weight number of 341. None of the samples contained the above-mentioned specific ions for aristolochic acid I, or their concentration was below the detection limit (0.02 μM) of our method.

**Figure 1:**
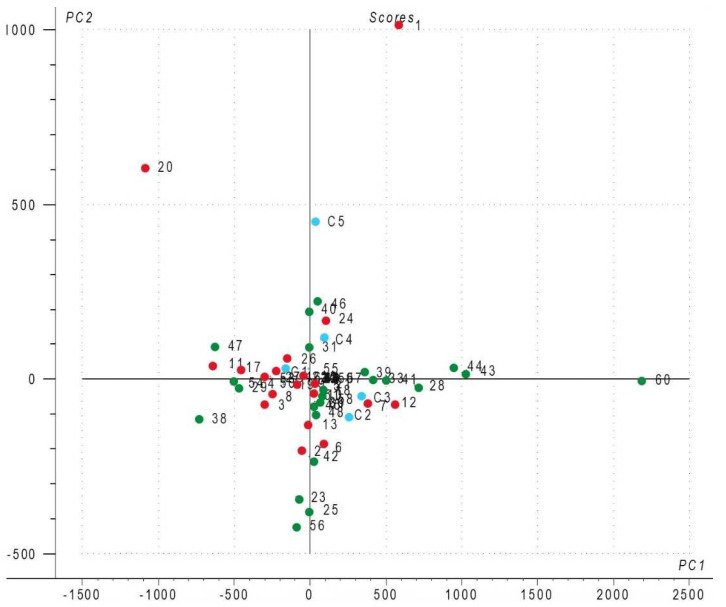
PC1 versus PC2 scores plot (all metabolites): red dots—BEN patients; green dots—healthy volunteers; blue dots—CKD patients.

Following the identification of compounds in the chromatograms, a matrix was constructed with all percentage areas corresponding to each of the patient/volunteer samples. Based on the data matrix, a first statistical analysis was performed with all cases (patients and volunteers) and all variables (urine metabolites) included. The distribution pattern of cases (Figure 1) is similar to the distribution pattern of Group 4 (Figure S4), whereas in the statistical analysis drug metabolites were excluded. For a better representation of the cases, PC1 versus PC2 scores plot were presented. Urine samples, represented by the three types of individuals (see code names in Section 2.3), are marked with colored dots in every score plot for better visualization, as follows: red dots represent BEN patients, green dots represent healthy controls, and blue dots represent CKD patients.

To obtain a specific distribution of cases and variables, six groups (Groups 1 to 6) were generated. PC1 versus PC2 score plots (Figures S1–S6) and loading plots (Figures S1–S6) are presented in the Supplementary Material. The classification of the six groups was based on the classes of metabolites: Group 1 includes only drug metabolites; Group 2 includes only phenolic compounds; Group 3 includes only hormone metabolites; Group 4 includes all metabolites without drug metabolites; Group 5 includes all metabolites without p-Cresol; and Group 6 includes only metabolites of BEN patients and their healthy family members.

In Group 1, urine samples and 12 metabolites resulting from the drug metabolism were considered. The following drugs were found in urine samples: brallobarbital, cyclobarbital, diazepam, amobarbital, phenobarbital, 3-(2-methylallyl) salicylic acid, antipyrine, and flufenamic acid. Some of the resulting compounds represent drug metabolites: ampyrone, phenylhydrazone, 4-(diethylamino) salicylaldehyde, 6H-1,4-diazepin-6-one, 2,3-dihydro-5,7-dimethyl-, and metacetamol. Ampyrone and flufenamic acid were the most frequent drugs found in 39 urine samples, 13 of them being present in BEN patients. The statistical model did not reveal any significant results when BEN patients were compared with CKD patients and the control group. BEN patients, marked with red dots, cluster in the middle and the right side of the score plot (Figure S1), meaning that those urine samples contain drug metabolites (Figure S1).

In Group 2, phenolic compounds were considered for statistical evaluation, since they form one of the main classes of urine metabolites. The statistical model included eight phenolic compounds and did not reveal any significant results when comparing BEN patients with CKD ones because of the limited number of CKD patients. One of the most common phenolic compounds is p-Cresol, present in 77% of all urine samples (Figure S2). Although p-Cresol was present in the urine of 80% (*n* = 20) of BEN patients versus 66% (*n* = 23) of the controls, because of the small number of samples, the statistical model did not reveal any significance. Only one subject (C3) (Figure S2), representing a patient with another CKD, had p-Cresol at the highest percentage level compared to other samples.

In Group 3, only hormone metabolites (*n* = 18) were included for statistical evaluation, these being present in 74% of urine samples (Figure S3). The resulting statistical model did not reveal any significant relevance when the three types of individual samples were compared. Most of the BEN patients appear in the right lower part of the score plot (Figure S3), meaning that these patients had more than five types of hormones in their urine samples. The most frequent hormones appearing in urine samples were: androst-5-ene-17-carbonitrile, 4-acetoxy-17-hydroxy; androst-2-en-17-one, an endogenous steroid; androstan-17-one, 3-hydroxy; 3-formyloxy-androst-11-en-17-one. Also, 9,10-Secocholesta-5,7,10(19)-triene-3,24,25-triol (calcitetrol), a hormonal form of vitamin D3, was found in one control sample.

In Group 4, drug metabolites were excluded from the statistical evaluation because these were not specific to the treatment of BEN or other chronic kidney diseases and most likely resulted from generic over-the-counter self-medication. BEN patients appear in two parts of the score plot (Figure S4), one group in the right lower part of the plot and the other group in the left part of the plot. Subject 60 (control subject from a BEN area) is in the right corner of the score plot (Figure S4); this urine sample contains several compounds, only two in a high percentage: squalene (11%) and cholesterol (75%) (Figure S4).

In Group 5, p-Cresol was excluded, since it was one of the most frequently encountered compounds in all types of urine samples (77%). Generally, this is one of the most common phenolic compounds present in urine samples, typically found at a high percentage in subjects with affected kidneys (Figure S5).

In Group 6, all metabolites of urine samples from family members of patients were considered. Ten families were identified as living in BEN areas, some of the members being BEN patients and others being healthy controls (code names are F followed by the number of the family (e.g., F1) and the number of the individual (e.g., F1-1). The following families have one member with BEN (F1; F2; F3; F6; F9), while other families include two members with BEN (F4; F5; F7). There are also two families with healthy controls (F8; F10). A statistical model without significance was obtained when urine samples of BEN patients and controls were compared. As can be observed, BEN patients appear at the right upper and lower parts of the score plot (Figure S6).

## 4. Discussion

In the research field of Balkan endemic nephropathy, this study is the first involving human urine samples collected from patients with BEN, volunteer controls, and patients with other types of CKDs. Upon extraction, the samples were analyzed using the simple and sensitive GC-MS technique. In BEN, as in AKI and CKD, normalization to urinary creatinine (UCr) concentration is not necessary when comparing across individuals with impaired renal function. The UCr concentration changes rapidly, leading to different creatinine generation rates [20]. Accordingly, interpretation of the kidney injury biomarker levels in our study was not performed by creatinine normalization. Because BEN patients are subject to hemodialysis three times a week, they exhibit high UCr excretion rate variability that could be misleading. Timed urine sample collection, as performed in this study, provides an estimate of biomarker excretion rate, which can be comparable from one individual to another [20].

The GC-MS technique was chosen as the method of analysis because even when limited to the detection of volatile organic compounds (VOCs), it is a non-invasive screening technique, and volatile metabolites from urine analysis result from different classes of compounds that could reveal specific disease patterns [21]. The mechanism for the generation of urinary VOCs is perturbed in physiological and pathological states, which include older age, dietary habits, environmental exposure, health and disease states, their origin being unclear [22]. Smith et al. (2008) have identified more than 200 volatiles in human urine, belonging to different chemical classes such as alcohols, ketones, hydrocarbons, pyrroles, furans, aldehydes, terpenes, sulphur-containing compounds (isocyanates, sulphides), and O- and N-heterocyclic compounds [23]. The resulted urinary VOCs in our study were also from the ketone, aldehyde, and hydrocarbon classes of compounds. These metabolic products result from the breakdown of both exogenous (foods, drinks, drugs, environmental contaminants), and endogenous (metabolites, bacterial byproducts) compounds [8]. The most frequent urinary VOCs obtained in the present study are ketones, which represent around 21% of all compounds, followed by hydrocarbons, alkanes, alkenes and alkynes, representing 16%. Other types of compounds found in urine samples are byproducts resulting from the fragmentation of other molecular structures, or represent precursors of other compounds or intermediates in the synthesis of other compounds (Table S2) [24,25,26,27,28,29,30]. Other compounds come from metabolism of tryptophan, aromatic amino acids, or dietary polyphenols, which are protein-bound uremic toxins [31]. Another compound found in our samples was hippuric acid, which was present in more than 30% of BEN patient urine samples compared to 14% of the control samples. Even though hippuric acid is known to exhibit elevated concentration in CKDs [31], our results showed similar average concentrations in BEN cases and controls. More than 50% of urinary compounds were derivatives of dietary origin: hippurates, indoles, phenols, polyamines [32].

The first classification for the statistical evaluation was made with drug metabolites resulting from self-medication, although none of the patients and volunteers disclosed specific treatments they were following at the time of sampling. Most of the main molecular ions were specific for the non-steroidal anti-inflammatory drug class. It is worth mentioning that the distribution pattern of the score plot from Figure 1 was similar to Figure S4. In Figure S4 drug metabolites were excluded from the statistical evaluation, meaning that they have no influence on the interpretation of the results; this confirms that their use is not included in a specific medication and has no apparent influence in the evolution of BEN disease.

A second classification was made for metabolites that contained phenolic groups. Phenolic compounds are considered typical urine biomarkers. The activity of gut microbiota through dietary polyphenols and aromatic amino acids fermentation leads to the presence of phenols in urine samples. Phenol derivatives result from the metabolism of tyrosine and phenylalanine through the intestinal microbiome activity. One of the main phenolic compounds is p-Cresol, which was found in 80% of BEN patients. This compound is slowly cleared by dialysis, and is accumulated in the kidneys, leading to inflammation and fibrosis [31]. Variations in the metabolites containing the active group phenyl- suggest that the gut microbiota could have been affected by the presence of aristolochic acid [33]. 

Another major class of compounds present in urine samples was hormones, which have endogenous and exogenous sources. Most hormone presence is due to hormonal medications. Most of the subjects were older than 45 (70%), and some of the female subjects were receiving estrogen replacement therapies at the time of sample collection. The most common hormonal metabolites detected in urine samples are found in the center of the loading plot (Figure S3).

The major aristolochic acid ions were searched based on the mass spectrum of the aristolochic acid I standard, but none of these molecular ions could be detected, probably because the subjects were not exposed to aristolochic acids around the time of urine sample collection, or because the concentrations were below the detection limit [34]. One of the most frequent routes of exposure to aristolochic acids is by ingestion of various herbal preparations that contain *Aristolochiaceae* plants [34]. In a recent review, Duan et al. (2018) describe some metabolomic technologies used in the evaluation of nephrotoxic effects of traditional Chinese medicine [35]. In a study of aristolochic acid nephrotoxicity in rats, urine samples were derivatized and analyzed using GC-MS and a PCA statistical evaluation. Only one biomarker corresponds to our investigations, namely, uric acid, the concentration of which is time- and dose-dependent on aristolochic acid-induced nephrotoxicity, and is also described as a “late metabolic biomarker”. The presence of uric acid and its increase in urine samples indicates that purine metabolism has been altered, and could also lead to other metabolic modifications associated with renal function impairment [36].

Other environmental toxins to which BEN patients are exposed, and which are considered cofactors in disease causation, are polycyclic aromatic hydrocarbons (PAHs), which contaminate groundwater supplies and have anthropogenic or geogenic sources such as Pliocene lignite deposits found in the vicinity of the endemic villages [37]. Although PAHs are ubiquitous in the environment, studies have confirmed their presence in less than 5% of the urine samples [38], which is in accordance with our results, in which the presence of two PAHs was found in around 8% of the urine samples. The two PAHs present in urine samples were 3′,8,8′-trimethoxy-3-piperidyl-2,2′-binaphthalene-1,1′,4,4′-tetrone and methanesulfonic acid, 17-cyano-10,13-dimethylhexadecahydrocyclopenta[a]phenanthren-17-yl ester. Their detection is due to the presence of cotinine and nicotine metabolites in subjects who were active cigarette smokers.

This study has some limitations because it presents preliminary results obtained from a limited number of patients. Because of the limited number of tested subjects, the PCA statistical method was chosen to reveal specific urinary biomarkers. There is a need for control samples from the population living in BEN areas, as well as urine samples taken from BEN patients at different timepoints. Furthermore, a continuous study over a longer period, and a complex epidemiological study associated with other biological biomarkers (urinary, fecal, blood, renal tissue samples), could provide better knowledge for BEN prevention. The chosen liquid-liquid extraction method without derivatization resulted in a limited number of volatile compounds, usually thermostable ones. Other limitations of this study include the limited incidence of BEN cases in Romania (only in the southwestern part of the country). Differences in urine sample concentration, using creatinine levels as a proxy, were absent. Other extraction techniques and analytical methods should be used to identify other types of urinary metabolites beside those obtained by the GC-MS method in order to generate a large dataset of compounds from all chemical compound classes. The generated results should be analyzed using univariate and multivariate statistical methods combined with artificial intelligence techniques, which could lead to robust models necessary to obtain specific and discriminative biomarkers [14]. An international collaboration with other BEN countries (Serbia, Bulgaria, Bosnia and Herzegovina, Croatia) could have a significant impact on health care economics and on reducing patient morbidity and mortality. National legislation could facilitate regular screening of the susceptible population (those living in the endemic areas) and could allow the obtained results to be introduced into a global database to facilitate positive diagnosis of the disease.

## 5. Conclusions

To the best of our knowledge, this study is the first to use urine metabolomics to discriminate among specific biomarkers in a limited number of individuals, including BEN patients, patients suffering from other types of CKD, and healthy individuals living in BEN areas. The liquid-liquid-extraction method was employed to extract a large panel of metabolites. The GC-MS analysis resulted in a panel of 262 urinary compounds, which seemed to be appropriate before statistical evaluation using the PCA technique. Although the statistical models generated using PCA did not reveal any significant results because of the limited number of samples of studied groups, it is worth mentioning that some key metabolites were found in a higher percentage and in a higher number of BEN samples than those found in the control group. The increased metabolites in urine BEN patients are phenolic compounds, especially p-Cresol, which implies an impairment of the renal filtration function. The discovery of p-Cresol as a marker involved in BEN pathology development could be used for early renal impairment diagnosis before the disease is clinically manifested. The excretion of dicarboxylic acids, cholesterol, and phenolic acids in the urine suggests severe oxidative stress processes. Other specific compounds for BEN urine samples that came to light after the PCA statistical evaluation were protein-bound uremic toxins with specific functional groups: indole, phenyl, phenol. These urinary metabolites provide a particular metabolomic signature when compared to normal urine samples, and could be used in a screening procedure to diagnose early forms of BEN in the population in endemic villages. Because of the limited number of CKD patients, no specific urinary metabolites were identified compared to BEN patients. So far, no studies have been performed using urinary analysis in search of specific biomarkers for early BEN diagnosis. All these methods, including extraction, chromatography, and statistics (LLE—GC-MS—PCA), could serve as primary noninvasive screening tools for generating preliminary data, leading to prompt decision making, which is necessary for further investigation of BEN patients.

## Figures and Tables

**Table 1:** Demographic characteristics of the study population.

Category	*n* (%)
Male	28 (43.1%)
Female	37 (56.9%)
Group categories of study population	
Healthy volunteers	35 (53.9%)
BEN patients	25 (38.5%)
CKD patients	5 (7.7%)
Rural population	60 (92.3%)
Urban population	5 (7.7%)
	Mean age ± standard deviation years
Age (mean ± standard deviation)	65.09 ± 2.16

## Data Availability

The authors confirm that the data supporting the findings of this study are available within the article and its Supplementary Materials.

## Supplementary Materials

The following supporting information can be downloaded at https://www.mdpi.com/article/10.3390/metabo13050609/s1. Table S1. Common urine metabolites and their origins; Table S2. By-products representing precursors of other compounds; intermediates in the synthesis process of some compounds; final products resulting from precursors and intermediates in the metabolic process; Figure S1. PC1 versus PC2 scores plot of Group 1 (drug metabolites) (red dots−BEN patients, green dots−healthy volunteers, blue dots−CKD patients); PC1 versus PC2 loading plot of Group 1 (drug metabolites); Figure S2. PC1 versus PC2 score plot of Group 2 (phenolic compounds) (red dots−BEN patients, green dots−healthy volunteers, blue dots−CKD patients); PC1 versus PC2 loading plot of Group 2 (phenolic compounds); Figure S3. PC1 versus PC2 score plot of Group 3 (hormone metabolites) (red dots−BEN patients, green dots−healthy volunteers, blue dots−CKD patients); PC1 versus PC2 loading plot of Group 3 (hormone metabolites); Figure S4. PC1 versus PC2 score plot of Group 4 (all metabolites without drug metabolites) (red dots−BEN patients, green dots−healthy volunteers, blue dots−CKD patients); PC1 versus PC2 loading plot of Group 4 (all metabolites without drug metabolites); Figure S5. PC1 versus PC2 score plot of Group 5 (all metabolites without p-Cresol) (red dots−BEN patients, green dots−healthy volunteers, blue dots−CKD patients); PC1 versus PC2 loading plot of Group 5 (all metabolites without p-Cresol); Figure S6. PC1 versus PC2 score plot of Group 6 (all metabolites of BEN patients and their healthy family members) (red dots−BEN patients, green dots−healthy volunteers, blue dots−CKD patients); PC1 versus PC2 loading plot of Group 6 (all metabolites of BEN patients and their healthy family members).
