# Supplementary material for: Urine Metabolomic Signature of People Diagnosed with Balkan Endemic Nephropathy and Other Types of Chronic Kidney Disease Compared with Healthy Subjects in Romania
Source: Metabolites. 2023 Apr 28;13(5):609. doi: 10.3390/metabo13050609 (PMC10221794; doi:10.3390/metabo13050609)
Supplement: Supplementary file 1 [file metabolites-13-00609-s001.zip › metabolites-2322659-supplementary.pdf]

Supplementary Material

Article

Urine Metabolomic Signature of People Diagnosed with Balkan Endemic Nephropathy and Other Types of Chronic Kidney Disease Compared with Healthy Subjects in Romania

Valentin L. Ordodi [1], Nicoleta G. Hădărugă [2], Daniel I. Hădărugă [1], Alexandra T. Lukinich-Gruia [3,*], Mihaela Mărgineanu [4], Călin A. Tatu [3,5] and Virgil Păunescu [3,5]

[1] Department of Applied Chemistry, Organic and Natural Compounds Engineering, Polytechnic University of Timisoara, Carol Telbisz 6, 300001 Timisoara, Romania; valentin.ordodi@upt.ro (V.L.O.); daniel.hadaruga@upt.ro (D.I.H.)

[2] Department of Food Science, Banat University of Agricultural Sciences and Veterinary Medicine Timisoara, Calea Aradului 119, 300645 Timisoara, Romania; nicoletahadaruga@usab-tm.ro

[3] Centre for Gene and Cellular Therapies in the Treatment of Cancer—OncoGen, Clinical County Hospital Timisoara, Blvd. Liviu Rebreanu 156, 300736 Timisoara, Romania; geomed88@gmail.com (C.A.T.); vpaunescu@umft.ro (V.P.)

[4] Dialysis Center Fresenius NephroCare, 220012 Drobeta-Turnu Severin, Romania

[5] Department of Functional Sciences, "Victor Babes" University of Medicine and Pharmacy Timisoara,
300041 Timisoara, Romania

* Correspondence: alexandra.gruia@hosptm.ro

Table S1. Common urine metabolites and their origins.

Origin of metabolite		Metabolite
**Exogenous metabolites**		
Drugs	Anti-inflammatory	- Metacetamol;<br>- Ampyrone;<br>- Antipyrine;<br>- Flufenamic acid;<br>- 3-(2-Methylallyl)salicylic acid;<br>- 4-(Diethylamino)salicylaldehyde;<br>- Chlorpheniramine;
	Sedative	- Amobarbital;<br>- Phenobarbital;<br>- Brallobarbital;<br>- Cyclobarbital;<br>- Diazepam;<br>- 6H-1,4-Diazepin-6-one, 2,3-dihydro-5,7-dimethyl, phenylhydrazone;
	Antibiotics	- Nalidixic acid
Environmental contaminants:		
Pesticides		- Pyrazone;
Phthalates		- Diisooctyl phthalate;<br>- Benzyl butyl phthalate;<br>- Diethyl Phthalate;
Terpenes, terpenoids		- p-Mentha-6,8-diene, 2-hydroperoxide;<br>- Cucurbitacin b, 23,24-dihydro-25-deacetoxy;<br>- Sulforaphane;<br>- Eugenol;<br>- Carvone;<br>- 8-Hydroxycarvotanacetone;<br>- Limonene;<br>- Orcinaldehyde;<br>- Carotol;<br>- 1-Methylene-spiro[4.5]decan-6-one;<br>- 5-Isopropyl-2,8-dimethyl-9-oxatricyclo[4.4.0.0(2,8)]decan-7-one;<br>- Ethyl chrysanthemate;<br>- Bicyclo[2,2,1]heptane-1-carboxylic acid, 7,7,0dimethyl-2-oxo;<br>- 3-Hydroxy-5a-methyldecahydro-3,9a-methano-2-benzazepin-1-one;<br>- 7-Methoxycoumarin/Ayaparin;<br>- 4-(2-Butyl)-cis-bicyclo[4.3.0]-2-nonen-8-one;<br>- Aromadendrene oxide-(2);<br>- Squalene;
Food and hygiene products:<br>Synthetic compounds<br>Flavoring agents in foods and cosmetic products		- α-Cyclocitral;<br>- gamma-Valerolactam;<br>- Methyldiethanolamine;<br>- Acetic acid, 2-(2,3-dihydro-2-imino-3-methyl-1-benzimidazolyl);<br>- p-Vinylguaiacol;<br>- o-Hydroxyacetophenone;

	- 10-Pentadecen-1-ol;
	- o-Aminoacetophenone;
	- Methylparaben;
	- Triclosan;
	- 3-tert-Butyl-4-hydroxyanisole;
	- Dimetridazole;
	- Cedryl propyl ether;
	- alpha-Tocopheryl acetate
Natural compounds	
Smoking byproducts	- Nicotine;
	- Cotinine;
Coffee, tea	- Caffeine;
	- 1,7-Dimethylxanthine, 9H-Xanthen-9-one, 3-hydroxy;
	- Dihydroxanthin;
	- Theophylline;
	- Theobromine;
Plants	- Vanillin;
	- Apocynine;
	- Allomatrine;
	- 24-Noroleana-4(23),12-diene, 3-methyl;
	- 1H-2,8a-Methanocyclopenta[a]cyclopropa[e]cyclodecen-11-one, 1a,2,5,5a,6,9,10,10a-octahydro-5,5a,6-trihydroxy-1,4-bis(hydroxymethyl)-1,7,9-trimethyl;
	- 2-Methyl-2-[4-methyl-6-(2,6,6-trimethyl-1-cyclohexenyl)-1,3,5-hexatrienyl]-1,3-dioxolane;
	- Tryptanthrine;
**Endogenous metabolites**	
Hormones, sterols	- Cholesterol;
	- Androstenolone;
	- Androsterone;
	- 9,10-Secocholesta-5,7,10(19)-triene-3,24,25-triol;
	- Androst-5-ene-17-carbonitrile, 4-acetoxy-17-hydroxy;
	- Androstane-3,16-diol;
	- Androst-16-en-3-ol;
	- Androstane-3,7,17-trione, cyclic 7-(1,2-ethanediyl acetal);
	- Androst-2-en-17-one;
	- Androst-5-ene-17-carbonitrile, 4-acetoxy-17-hydroxy;
	- 6-Hydroxy-17-oxo-4-propyl-3,4-seco-5á-androstan-3-oic acid;
	- 5-(2-Butenyl)-17-oxo-4-nor-3,5-seco-5à-androstan-3-oic acid, methyl ester;
	- Spiro[androst-5-ene-17,1'-cyclobutan]-2'-one, 3-hydroxy;
	- Androstan-17-one, 3-hydroxy;
	- Androst-11-en-17-one, 3-formyloxy;
	- Androstane-11,17-dione,3-[(trimethylsilyl)oxy]-,17-[O-(phenylmethyl)oxime];
	- Ergosta-5,22-dien-3-ol, acetate;
	- Epiandrosterone;

	- Pregnan-20-one, 3-(acetyloxy);<br>- 5alpha-Pregnanediol;<br>- Lanosta-7,9(11),20-triene-3á,18-diol, diacetate;<br>- Pregnan-20-one, 3,17-dihydroxy;
Phenols	- p-Cresol;<br>- Phenol, 3-methoxy;<br>- Phenol, 4-(2-propenyl)-, acetate;<br>- Butylphen;<br>- Phenol, 2,6-dimethoxy-;<br>- Phenol, 4,6-di(1,1-dimethylethyl)-2-methyl;<br>- Antiarol;<br>- Phenol, 2,6-bis(1,1-dimethylethyl)-4-ethyl;
Fatty acids and Esters	- 5,8,11,14-Eicosatetraenoic acid, phenylmethyl ester;<br>- Nonanoic acid;<br>- 2-(2-Naphthyl)-2-hydroxypropanoic acid;<br>- Heptanoic acid, 2-methyl-, methyl ester;<br>- Propanoic acid, 3-chloro-, 4-formylphenyl ester;<br>- Propionic acid, 3-cyclohexyl-, 4-methoxycarbonylphenyl ester;<br>- Acetic acid, 8-acetyl-2,3,-dihydro-1,4-benzodioxin-5-yl ester;<br>- Acetic acid, 2-(2,2,6-trimethyl-7-oxa-bicyclo[4.1.0]hept-1-yl)-propenyl ester;<br>- Tetradecanoic acid;<br>- 1H-Indole-3-acetic acid, ethyl ester;<br>- 11-Hexadecenoic acid;<br>- Phosphoric acid, dimethyl(4-methoxy-3-t-butylphenyl) ester;<br>- Hexadecanoic acid/Oleic acid;<br>- 2-(4-Hydroxy-4-methyl-tetrahydro-pyran-3-ylamino)-3-(1H-indol-2-yl)-propionic acid;<br>- 9,12-Octadecadienoic acid;<br>- Oleic Acid;<br>- Hexadecanoic acid, butyl ester;<br>- 9,12,15-Octadecatrienoic acid, 2-(acetyloxy)-1-[(acetyloxy)methyl]ethyl ester;<br>- Octadecanoic acid;<br>- 9,12-Octadecadienoic acid;<br>- Methyl-5,11,14,17-eicosatetraenoate;<br>- 5,8,11,14-Eicosatetraenoic acid, methyl ester;<br>- Stearic acid, 2-(1-octadecenyloxy)ethyl ester;<br>- Cholesterol margarate;<br>- Methanesulfonic acid, 17-cyano-10,13-dimethylhexadecahydrocyclopenta[a]phenanthren-17-yl ester;

Table S2. By-products representing precursors of other compounds; intermediates in the synthesis process of some compounds; final products resulted from precursors and intermediates in the metabolic process.

Compound	Precursor of compound	Reference
Precursors		
Formanilide	- additive in rubber products;<br>- common synthetic intermediate;<br>- precursor to the fungicide mepanipyrim;	[1]
Intermediates		
Benzoic Acid	- occurs naturally in many plants;<br>- intermediate in the biosynthesis of secondary metabolites;<br>- precursor for the industrial synthesis of other organic substances;<br>- food preservative, represented by the E numbers E210, E211, E212, E213.	[2]
para-Cresol	- intermediate in the production of other chemicals;<br>- used in the production of antioxidants, butylated hydroxytoluene (BHT);<br>- produced by bacterial fermentation of protein in the human large intestine;<br>- excreted in the urine;<br>- constituent of tobacco smoke;	[3]
3-Methoxy-phenol	- derives from resorcinol;<br>- food additive with the E320;<br>- antioxidant and preservative in food, food packaging, cosmetics;<br>- used in medicines (e.g. Vitamin D3, isotretinoin, lovastatin, simvastatin).	[4]
Products of the metabolic breakdown		
Isatin	- metabolic derivative of adrenaline;	[5]
Uric acid	- product of the metabolic breakdown of purine nucleotides;	[6]
Final products		
Hippuric acid	- formed from the combination of benzoic acid and glycine;<br>- its levels rise with the consumption of phenolic compounds (e.g. fruit juice, tea);<br>- phenols are converted to benzoic acid, then to hippuric acid and excreted in urine;	[7]

References [24, 25, 26, 27, 28, 29, 30] are cited in the Supplementary Materials

1. Yu, H.; Wu, Z.; Wei, Z.; Zhai, Y.; Ru, S.; Zhao, Q.; Wang, J.; Han, S.; Wei, Y. N-Formylation of Amines Using Methanol as a Potential Formyl Carrier by a Reusable Chromium Catalyst. *Commun Chem* **2019**, *2*, 15, doi:10.1038/s42004-019-0109-4.
2. Maki, T.; Takeda, K. Benzoic Acid and Derivatives. In *Ullmann's Encyclopedia of Industrial Chemistry*; Wiley, 2000 ISBN 978-3-527-30385-4.
3. Fiege, H. Cresols and Xylenols. In *Ullmann's Encyclopedia of Industrial Chemistry*; Wiley-VCH Verlag GmbH & Co. KGaA, Ed.; Wiley-VCH Verlag GmbH & Co. KGaA: Weinheim, Germany, 2000; p. a08_025 ISBN 978-3-527-30673-2.
4. EFSA Panel on Food Additives and Nutrient Sources added to Food (ANS) Scientific Opinion on the Re-Evaluation of Butylated Hydroxyanisole – BHA (E 320) as a Food Additive. *EFSA Journal* **2011**, *9*, 2392, doi:10.2903/j.efsa.2011.2392.
5. Chiyanzu, I.; Hansell, E.; Gut, J.; Rosenthal, P.J.; McKerrow, J.H.; Chibale, K. Synthesis and Evaluation of Isatins and Thiosemicarbazone Derivatives against Cruzain, Falcipain-2 and Rhodesain. *Bioorganic & Medicinal Chemistry Letters* **2003**, *13*, 3527–3530, doi:https://doi.org/10.1016/S0960-894X(03)00756-X.

6. Heinig, M.; Johnson, R.J. Role of Uric Acid in Hypertension, Renal Disease, and Metabolic Syndrome. *Cleveland Clinic Journal of Medicine* **2006**, *73*, 1059–1064, doi:10.3949/ccjm.73.12.1059.
7. Duranton, F.; Cohen, G.; De Smet, R.; Rodriguez, M.; Jankowski, J.; Vanholder, R.; Argiles, A. Normal and Pathologic Concentrations of Uremic Toxins. *J Am Soc Nephrol* **2012**, *23*, 1258–1270, doi:10.1681/ASN.2011121175.

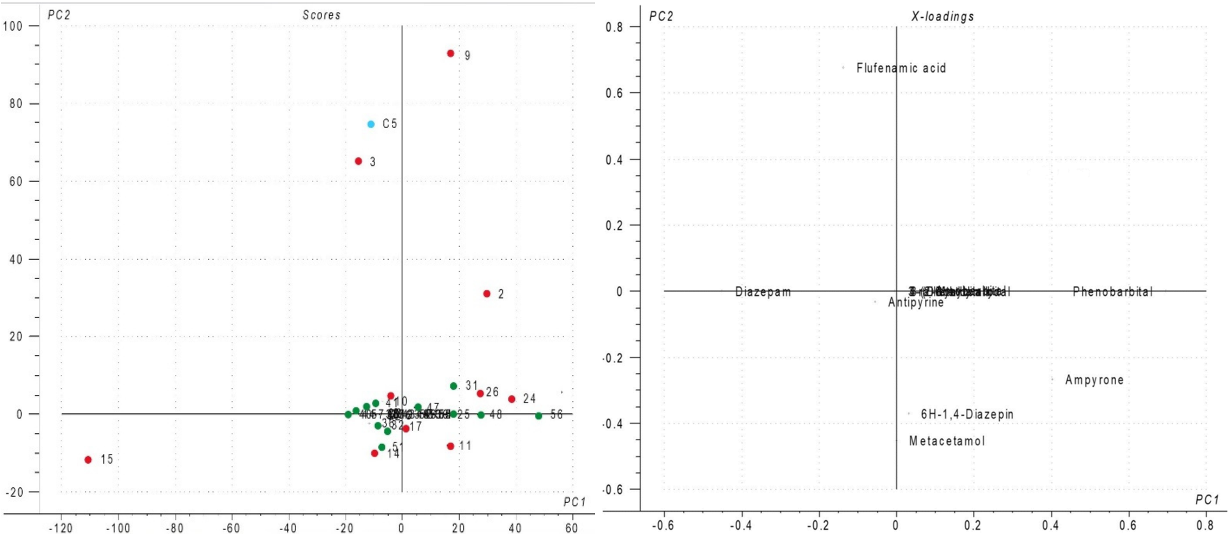

Figure S1. PC1 versus PC2 scores plot of Group 1 (drug metabolites) (red dots−BEN patients, green dots−healthy volunteers, blue dots−CKD patients); PC1 versus PC2 loadings plot of Group 1 (drug metabolites)

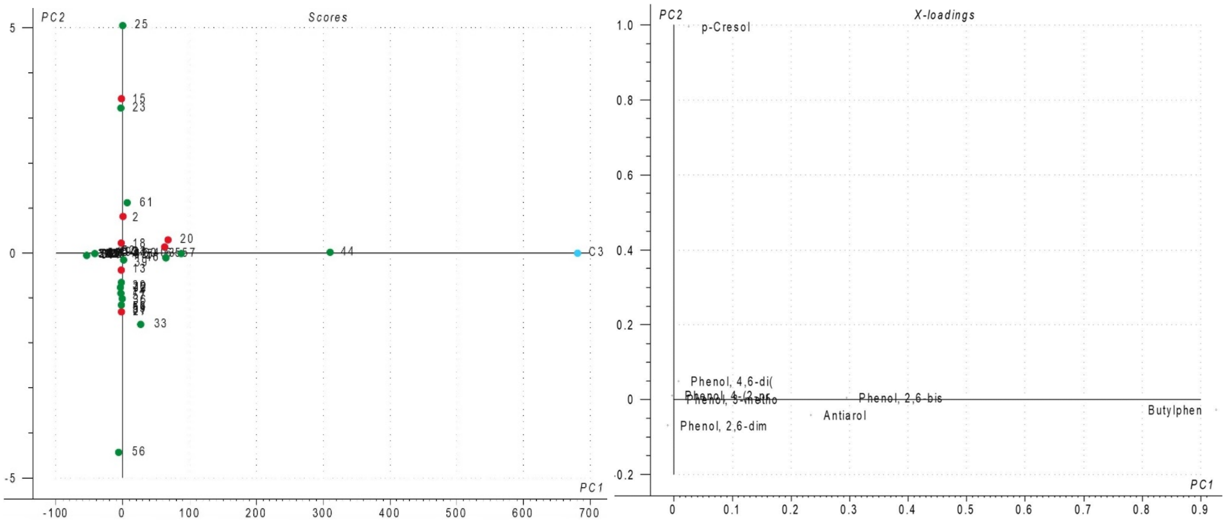

Figure S2. PC1 versus PC2 scores plot of Group 2 (phenolic compounds) (red dots−BEN patients, green dots−healthy volunteers, blue dots−CKD patients); PC1 versus PC2 loadings plot of Group 2 (phenolic compounds)

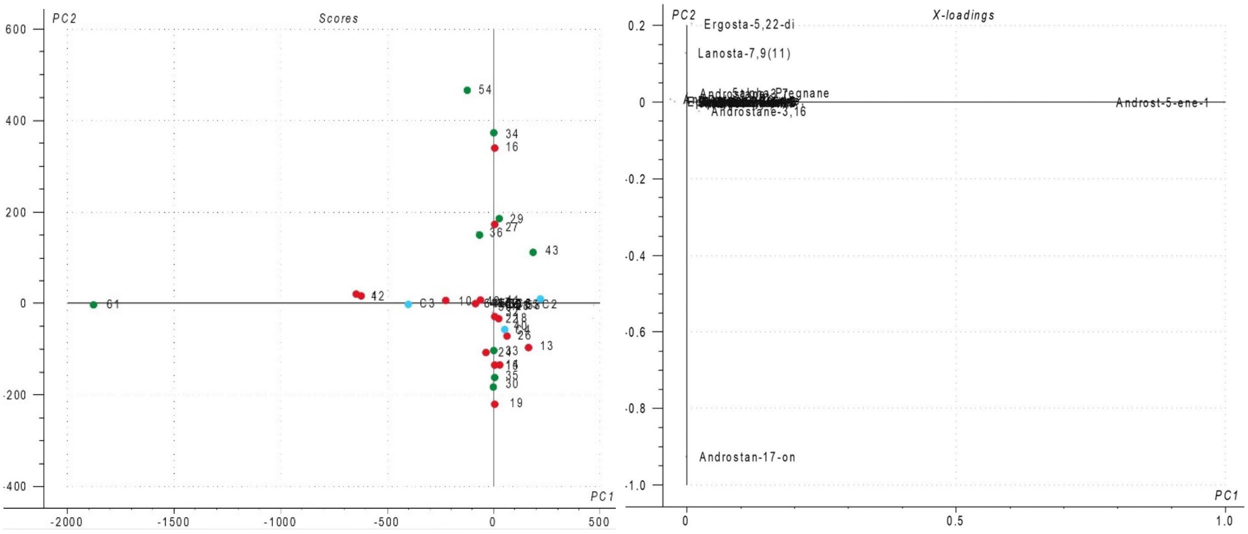

Figure S3. PC1 versus PC2 scores plot of Group 3 (hormone metabolites) (red dots−BEN patients, green dots−healthy volunteers, blue dots−CKD patients); PC1 versus PC2 loadings plot of Group 3 (hormone metabolites)

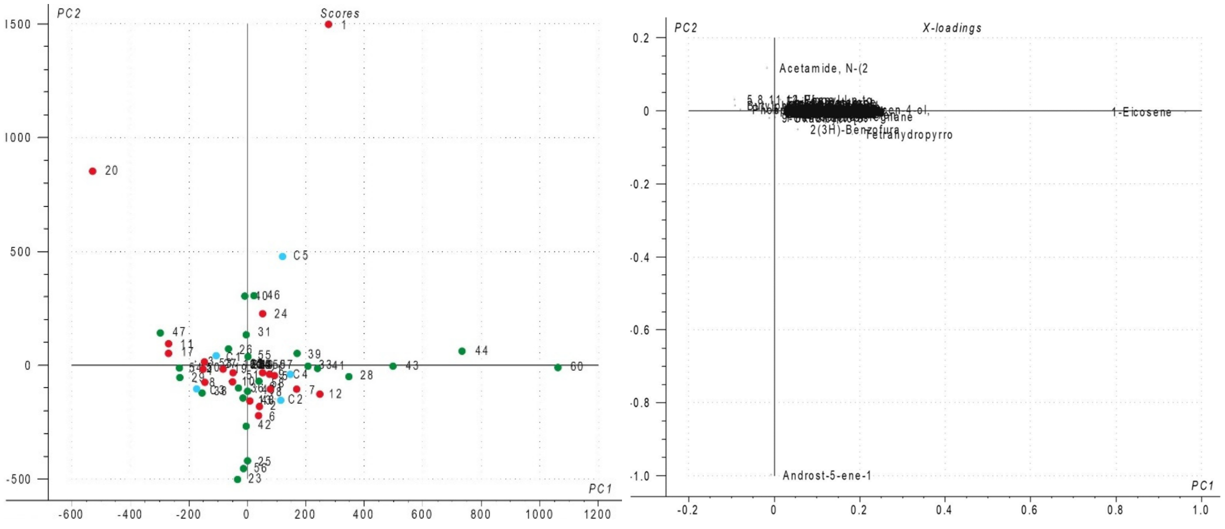

Figure S4. PC1 versus PC2 scores plot of Group 4 (all metabolites without drug metabolites) (red dots−BEN patients, green dots−healthy volunteers, blue dots−CKD patients); PC1 versus PC2 loadings plot of Group 4 (all metabolites without drug metabolites)

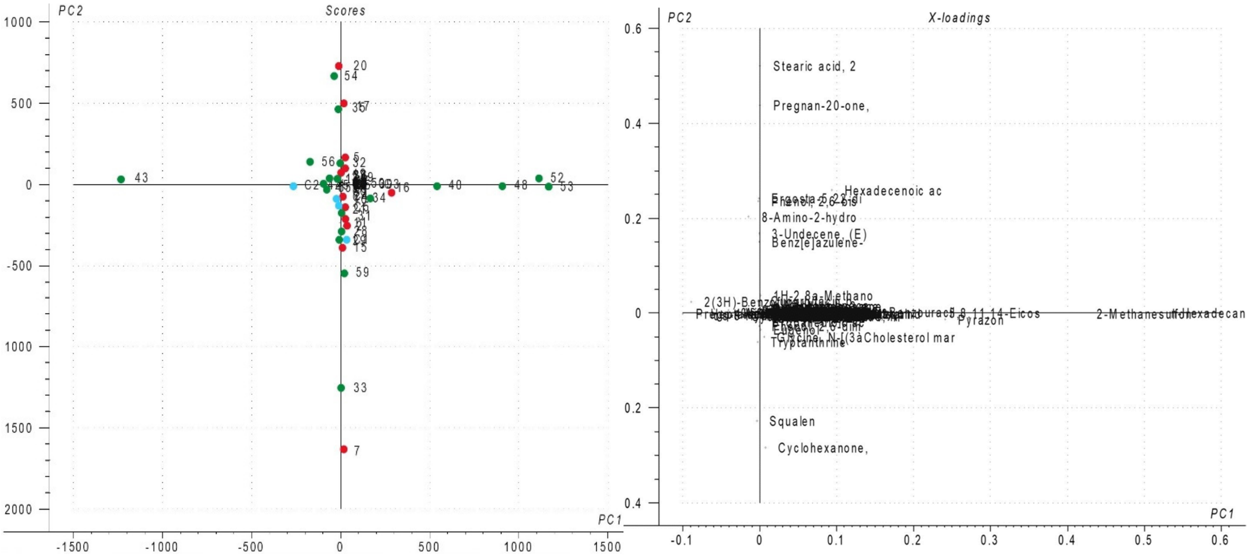

Figure S5. PC1 versus PC2 scores plot of Group 5 (all metabolites without p-Cresol) (red dots−BEN patients, green dots−healthy volunteers, blue dots−CKD patients); PC1 versus PC2 loadings plot of Group 5 (all metabolites without p-Cresol)

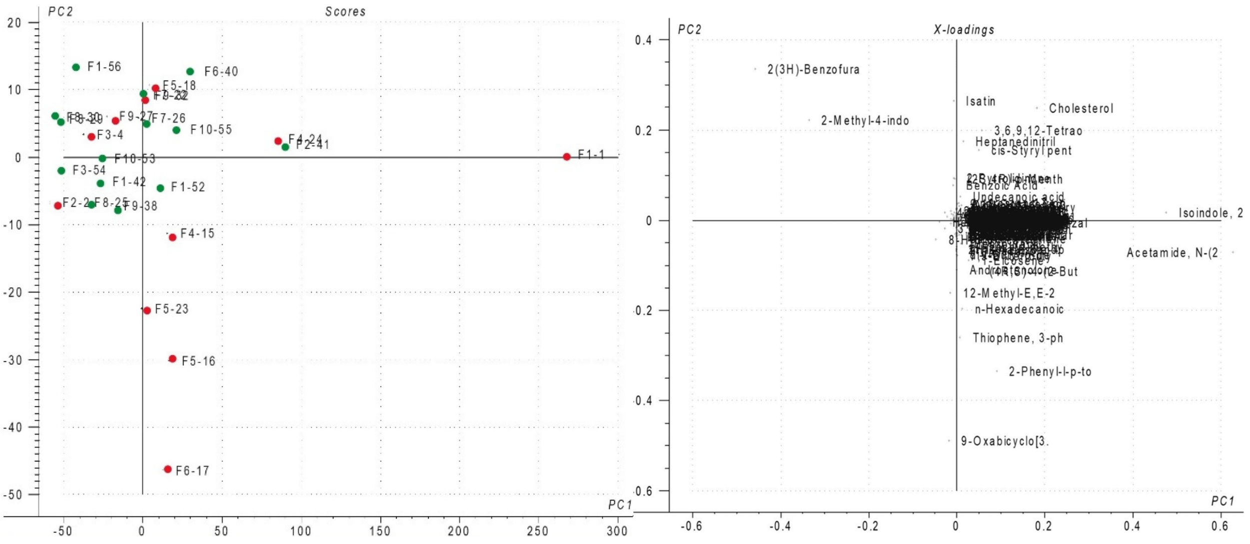

Figure S6. PC1 versus PC2 scores plot of Group 6 (all metabolites of BEN patients and their healthy family members) (red dots−BEN patients, green dots−healthy volunteers, blue dots−CKD patients); PC1 versus PC2 loadings plot of Group 6 (all metabolites of BEN patients and their healthy family members)
